# Assessing Health-Related Quality of Life of Chinese Adults in Heilongjiang Using EQ-5D-3L

**DOI:** 10.3390/ijerph14030224

**Published:** 2017-02-23

**Authors:** Weidong Huang, Hongjuan Yu, Chaojie Liu, Guoxiang Liu, Qunhong Wu, Jin Zhou, Xin Zhang, Xiaowen Zhao, Linmei Shi, Xiaoxue Xu

**Affiliations:** 1 School of Health Management, Harbin Medical University, 157 Baojian Road, Nangang District, Harbin 150086, China; weidong218@126.com (W.H.); zhangxinzhx0801@126.com (X.Z.); mary0822@163.com (X.Z.); slm_jl@163.com (L.S.); soar0865@sina.com (X.X.); 2 Department of Hematology, The First Affiliated Hospital of Harbin Medical University, Harbin 150001, China; yuhongjuan2008@163.com; 3 School of Psychology and Public Health, La Trobe University, Melbourne 3086, Australia; c.liu@latrobe.edu.au

**Keywords:** EQ-5D-3L, health-related quality of life, China

## Abstract

This study aimed to assess health-related quality of life (HRQOL) of Heilongjiang adult populations by using the EuroQol five-dimension three-level (EQ-5D-3L) questionnaire and to identify factors associated with HRQOL. Data from the National Health Services Survey (NHSS) 2008 in Heilongjiang province were obtained. Results of EQ-5D-3L questionnaires completed by 11,523 adult respondents (18 years or older) were converted to health index scores using a recently developed Chinese value set. Multivariate linear regression and logistic regression models were established to determine demographic, socioeconomic, health, and lifestyle factors that were associated with HRQOL and reported problems in the five dimensions of EQ-5D-3L. The Heilongjiang population had a mean EQ-5D-3L index score of 0.959. Lower EQ-5D-3L index scores were associated with older age, lower levels of education, chronic conditions, temporary accommodation, poverty, unemployment, and lack of regular physical activities. Older respondents and those who were unemployed, had chronic conditions, and lived in poverty were more likely to report problems in all of the five health dimensions. Higher educational attainment was associated with lower odds of reporting health problems in mobility, pain/discomfort, and anxiety/depression. Low socioeconomic status is associated with poor HRQOL. Regional population norms for EQ-5D-3L are needed for health economic studies due to great socioeconomic disparities across regions in China. Overall, the Heilongjiang population has a similar level of HRQOL compared with the national average.

## 1. Introduction

Cost-utility analysis (CUA) has been widely used in health technology assessment and health policy decisions [1,2]. In economics, utility is a measure of preference over certain goods, conditions, and services. In health, utility indicates people’s preference over a range of health conditions. Usually, an ill condition is placed on a utility scale on which 0.0 corresponds to death and 1.0 corresponds to full health (health states considered worse than death can be represented by negative values) [3].

Health utility can be estimated by using time trade-off (TTO), standard gambling (SG), and visual analogue scale (VAS) approaches [4]. It can be based on individual preference or social preference. Although there is ongoing debate about which approaches and methods should be adopted, most health economic studies have used social preference based on the argument that social policy and interventions have an impact on everybody in society [5,6]. Over the past few decades, several health related quality of life (HRQOL) assessment instruments have been developed, which can serve as a self-classifier for health [4,7]. The health status classified by the HRQOL instruments can be converted to a proxy health utility score using a predefined value set (social preference). For example, the EuroQol five-dimension (EQ-5D) [8,9], the Health Utilities Index (HUI) [10,11], and the Short Form six-dimension (SF-6D) survey [12,13,14] have become widely accepted in health utility studies. These are generic instruments that do not target any specific disease and thus can be used on the general public as well as people with various health conditions [15,16]. The EQ-5D (EuroQoL) is perhaps the most commonly used by researchers [17] and governmental agencies [18]. 

This study aimed to assess HRQOL of Heilongjiang adult populations using EQ-5D-3L and to identify factors associated with HRQOL. In mainland China, although the EQ-5D-3L has been validated [19,20,21] and recommended by the *Chinese Guidelines for Pharmacoeconomic Evaluations 2011* for use in health technology assessment [22], its population value set (social preference) has only recently been developed using a TTO approach (in 2014) [23] and VAS approach (in 2015) [24]. Past studies in China either used value sets of other countries to convert HRQOL results into a proxy utility score (called EQ-5D-3L index score) or restricted results to descriptions of reported HRQOL problems [24,25,26,27,28]. 

In public health, implications of the EQ-5D-3L index score are often interpreted against population norms (average scores of a population), which are benchmarks for evaluating population health care outcomes and health equality [29,30]. Population norms for the EQ-5D-3L index scores have been reported in the USA [31], Switzerland [32], Demark [33], Portugal [34], Australia [35], Taiwan [36], Singapore [37,38], Sri Lanka [39], and Japan [40,41]. No population norms for the EQ-5D-3L index scores have been reported in China.

## 2. Methods

### 2.1. Study Design and Data Collection

Data were derived from the fourth National Health Services Survey (NHSS) 2008 in Heilongjiang province, northeast of China. Heilongjiang, with a population of 38.33 million, ranks in the middle range of income among all provinces in China in terms of per capita GDP.

The 2008 NHSS adopted a multistage stratified random sampling strategy (details of the NHSS design have been published [42]). A total of 13 cities and counties in Heilongjiang were selected to participate in the survey. Within each selected city or county, eight communities/villages were chosen considering diversities in geographic location and economic status. Subsequently, 33 households in each community/village were randomly selected.

A questionnaire was administered to the selected participants through face-to-face interviews. The interviewers were recruited from local health workers. They were trained by a supervisor from their respective city/county who attended the NHSS training workshop organized by the Ministry of Health (MoH). The interviewers visited the households of the selected participants, and completed the questionnaire through interviewing the household members. The city/county supervisors checked the completeness of the returned questionnaires at the end of each day. Any missing data was collected by the interviewers through a second visit to the households.

A total of 15,875 individuals (from 5530 households) in Heilongjiang province completed the survey in 2008, among whom 13,183 were 18 years or older. About 99.5% (13,121) of adult respondents provided complete data, amongst whom 1598 (12.18%) used surrogate respondents and were excluded from the data analyses. This resulted in a final sample of 11,523 for the purpose of this study.

### 2.2. Instruments 

The 2008 NHSS questionnaire was developed by the MoH, collecting the demographic (e.g., age, sex, ethnicity) and socioeconomic (e.g., residency, marital status, educational attainment, employment, income, housing, and health insurance) data of the participants, health status including chronic conditions, and lifestyle (e.g., regular weekly physical activities) of the participants.

The 2008 NHSS questionnaire also included the Chinese version of EQ-5D-3L. The EQ-5D-3L is a self-administered paper-based questionnaire developed by the EuroQol Group [43]. It measures five dimensions of health: mobility, self-care, usual activities, pain/discomfort, and anxiety/depression; as well as overall health rated on a VAS. The EQ-5D-3L contains three levels of rating on the five health dimensions, corresponding to “no problems”, “some problems”, and “extreme problems”. This allows for 3^5^ (i.e., 243) possible combinations of health conditions [43,44]. The VAS scores ranged from 0 (worst health) to 100 (best health). The EQ-5D-3L has been widely accepted by various authorities for CUA [8], including the National Institute for Health and Clinical Excellence (NICE) in the UK [18]. 

### 2.3. Data Analysis

HRQOL results measured by the EQ-5D-3L were converted to an index score (a proxy health utility score) using the China TTO value set which ranges from −0.149 to 1.00 [23]. A negative value represents a health condition worse than being dead [23]. There are two population value sets available for the EQ-5D-3L in China: one developed by Liu et al. [23] using the TTO approach; another one developed by Sun et al. [24] using VAS. Although none of these methods is recognized as being the standard measure for valuing health in CUA [4,45], VAS is often considered to be inferior to TTO as it is not a choice-based method and the values are not anchored between dead and full health [4,6]. Furthermore, the *Chinese Guidelines for Pharmacoeconomic Evaluations 2015* [46] recommend TTO as a preferred method.

In line with recommendations of Kivits et al. [47], potential factors associated with HRQOL were classified into demographic (age, sex, ethnicity), socioeconomic (residency, marital status, educational attainment, employment, income, housing, and health insurance), and health (chronic conditions) variables. We also added regular weekly physical activities (≥1 per week moderate or vigorous exercises), a lifestyle indicator, into the analyses, which has been commonly used in HRQOL studies [41].

We categorized age into six groups (18–29, 30–39, 40–49, 50–59, 60–69, and ≥70 years); ethnicity into two groups (Han and others). The categorization of residency, marital status, educational attainments, employment, and housing status followed the original design of the NHSS questionnaire (Table 1). The NHSS did not collect reliable income data. As a result, we used officially recorded poverty data (yes or no) as a proxy indicator of income. The thresholds of the poverty line were defined by local governments and varied with local standards of living. Housing status was also associated with wealth: wealthier people were more likely to reside in a flat/apartment; whereas the poorest often stayed in an old house built from mudbricks.

In this study, health needs were measured by chronic conditions, which included hyperlipidemia, hypertension, heart disease, stroke, liver disease, diabetes mellitus, respiratory disease, renal disease, and gastrointestinal disease. A chronic condition was defined as a condition diagnosed by a doctor, for which either the symptoms persisted or relevant medical treatment continued over the past six months [24,38,39]. It was recorded as “yes” when a respondent had one or more of the chronic conditions.

We performed bivariate analyses first to examine the associations between the above factors and the HRQOL of respondents measured by the EQ-5D-3L. Mean EQ-5D-3L index scores and VAS scores were presented. Those scores were compared among the respondents with different characteristics using either student *t* tests (for two categories: sex, ethnicity, residence, officially recorded poverty, health insurance, chronic conditions, and regular weekly physical activities) or one-way ANOVA (for more than two categories: age, education, housing, marital status, and employment). In addition, we also performed non-parametric tests (Mann-Whitney U tests for two categories or Kruskal-Wallis tests for more than two categories) to examine differences in EQ-5D-3L index scores of the respondents because the distribution of data was skewed. To better understand the health problems experienced by the respondents, the percentage of people reporting any problem (some problems or extreme problems) in each dimension was calculated and x^2^ tests were performed to determine the statistical significance of the difference between groups in the percentage of reported problems.

We then performed multivariate analyses to determine the effect size of the HRQOL-associated factors after controlling for the influence of others. Multivariate linear regression models using enter method based on ordinary least square (OLS) approach were established to examine the statistical mean differences in the EQ-5D-3L index scores among respondents with different characteristics. Because ceiling effects (a level at which no further improvement can be demonstrated) are a common problem of EQ-5D-3L index scores, we also performed Tobit regression analyses, which produced consistent results as those of OLS (see Supplementary File). In addition, logistic regression models were developed with the five health dimensions as dependent variables (0 = no problem, 1 = some/extreme problems). Dummy variables were created for all of the independent variables in the modeling.

The statistical analyses were carried out using the Statistical Product and Service Solutions (SPSS) or STATA software. Statistical significance was set at 0.05 using two-sided tests.

This study was approved by the Ethics Committee of Harbin Medical University (Project Identification Code: HMUIRB2014012).

## 3. Results

### 3.1. Characteristics of Respondents 

The respondents had a mean age of 45.8 years: 48.8% were men and 95.2% were Han ethnicity. About 60% of respondents resided in rural areas. Twelve percent of respondents lived in a household officially recorded as in “poverty”. Most (74.2%) respondents were covered by health insurance. More than 21% of respondents reported diagnosed chronic conditions over the past six months. Less than 20% of respondents reported doing weekly physical activities regularly (Table 1).

### 3.2. Bivariate Analyses

The respondents obtained a mean EQ-5D-3L index score of 0.959 and a mean VAS score of 79.60. About 85% of respondents had the highest possible EQ-5D-3L index score, compared with 12.51% who rated 100 in VAS. The EQ-5D-3L index scores varied with the demographic and socioeconomic characteristics of respondents, except for ethnicity (*p >* 0.05). Older age (*p <* 0.001), female gender (*p <* 0.05), lower levels of education (*p <* 0.001), being widowed (*p <* 0.001), lack of insurance coverage (*p <* 0.001), and unemployment (*p <* 0.001) were associated with lower EQ-5D-3L index scores. Those who did not have permanent accommodation, lived in poverty, and had chronic conditions also had lower EQ-5D-3L index scores than their counterparts (*p <* 0.05). Although the respondents who did regular weekly physical activities had higher EQ-5D-3L index scores than those who did not, the difference was statistically insignificant (*p >* 0.05). The analyses of VAS scores generated consistent results as those of EQ-5D-3L index scores.

Pain/discomfort was the most frequently reported problem (12.1%), followed by anxiety/depression (7.2%), whereas self-care (3.6%) was the least frequently reported problem. Women were more likely to report problems in pain/discomfort (13.6%) and anxiety/depression (8.2%) than men (*p <* 0.05). Compared with respondents of Han ethnicity, the minority ethnic group was more likely to report problems in mobility (8.7%), self-care (5.3%), usual activities (7.4%), and pain/discomfort (13.8%) (*p <* 0.05). Those who lived in urban areas, were older, widowed, had lower levels of education, did not have permanent accommodation, lived in poverty, had no insurance coverage, suffered from chronic conditions, and did not do regular weekly physical activities were more likely to report problems in all five health dimensions (*p <* 0.05). The unemployed respondents were more likely to report problems in mobility, self-care, usual activities, and pain/discomfort, compared with the retired who were more likely to report problems in anxiety/depression (*p <* 0.05) (Table 2).

### 3.3. Multivariate Analyses

The logistic regression models revealed that those with a chronic condition had significantly higher odds (odds ratio (OR) ranging from 5.6 to 7.6) of reporting health problems. Women were less likely (OR ranging from 0.6 to 0.7) than men to report problems in mobility, self-care, and usual activities. Older age was associated with a higher likelihood of reporting health problems in all of the five health dimensions.

After adjustment of demographic and chronic conditions, however, lifestyle and socioeconomic factors remained significantly associated with self-reported health problems in EQ-5D-3L. Rural residents had slightly higher odds of reporting problems in mobility (OR = 1.6) and usual activities (OR = 1.8). The odds of reporting health problems decreased with educational attainment, with those who were college educated having an OR of 0.3–0.6 compared with those with less than primary school education. The respondents who fell into the government recorded category of poverty had an OR of 2.0–2.6 for reporting health problems compared with those without poverty. Respondents who did not have permanent accommodation had an OR of 2.4–3.2 for reporting problems in usual activities, pain/discomfort, and anxiety/depression compared with those who owned an apartment. The unemployed had higher odds of reporting health problems (OR = 1.6–2.7) than their employed counterparts. However, no associations between health insurance coverage, marital status, and self-reported health problems were found.

The logistic regression models also demonstrated associations between self-reported health problems and regular physical activities. Those who engaged in regular weekly physical activities had lower odds (OR = 0.3–0.8) of reporting health problems in mobility, self-care, usual activities, and anxiety/depression.

The multivariate linear regression analysis revealed that lower EQ-5D-3L index scores were associated with older age, lower levels of education, chronic conditions, temporary accommodation, poverty, unemployment, and lack of regular physical activities (Table 3). However, sex, residence, and health insurance were no longer statistically significant in predicting the EQ-5D-3L index scores.

## 4. Discussion

The Heilongjiang population has an average level of HRQOL in comparison with other populations. Overall, the Heilongjiang population had an average of EQ-5D-3L index score of 0.959, similar to the Singapore population norms (0.95) [37,38] and Taiwan population norms (0.946) [36], but higher than the norms reported in the USA (0.87) [31], Demark (0.889) [33], Sri Lanka (0.85) [39], and Japan (0.877) [40,41]. The mean VAS score of the Heilongjiang population (79.60) is only slightly lower than the national average (80.12) of China [24]. Pain/discomfort was the most frequently reported problem in our study, which is consistent with findings of EQ-5D studies undertaken elsewhere [20,49,50,51]. About 12% of our study participants reported pain/discomfort, similar to the national average [24]. However, it is much lower compared with those reported in Poland (40%) [50], Greece (43.5%) [51], the USA (40.8%) [20], and Sweden(44.3%) [49]. Previous studies showed that Asian populations are more likely to report better health status and demonstrate ceiling effects than Europeans in health utility studies, possibly due to cultural reasons [24,52,53]. The health problems experienced by a population may also be shaped by their demographic and socioeconomic structure [24,54,55].

The bivariate analyses found that women (13.6%) reported more problems in pain/discomfort than men (10.5%), although the multivariate logical regression analysis did not reach a statistical significance with 95% confidence interval (CI) of OR for the association ranging from 1.0 to 1.3. Similar results were also found in studies conducted elsewhere in China [24] and other countries [49,50,51]. A considerable amount of evidence from both laboratory research and clinical studies has proven the existence of gender differences in the experience of pain. Women appear to be at an increased risk of experiencing pain-related symptoms [56,57].

Similar to other studies [31,33,37,39,40,41], our bivariate and multivariate analyses also found that EQ-5D-3L index and VAS scores decline with age. Older people were more likely to experience problems in all of the five domains of EQ-5D-3L than their younger counterparts. The EQ-5D-3L index scores of those of 50 years and older dropped to a level of below average. Aging presents a great challenge to the world, both in developed and developing nations [58]. China turned into an ageing society in 1999. The sixth population census in 2010 showed that 13.26% of China’s population were 60 years and older. Those who are 65 years and older account for 8.87% of the total population [59]. With increased life span and aging populations, more people are now living with ill health, in particular, chronic non-communicable diseases (NCDs). NCDs have become a leading cause of death globally [60,61]. In China, it was estimated that NCDs account for 82.5% of the total deaths [62]. The regression models established in this study show that “chronic conditions” is the most significant single variable predicting EQ-5D-3L index scores (Beta = −0.09) and reported health problems (with an OR ranging from 5.6 to 7.6). It is important to note, however, that people’s preference can change, especially in countries like China where the recent economic miracle has been accompanied with dramatic lifestyle and cultural shifts. Parents who have only one child under China’s family planning policy have been widely exposed to western culture and values and they will become “elderly” in the next one or two decades. This will not only change the demographic landscape, but will also impose significant implications on people’s value on health. The value set for EQ-5D-3L needs to be updated on a regular basis.

Socioeconomic inequality in HRQOL is evident in both bivariate and multivariate analyses. People with no more than primary school education have significantly lower (below average) EQ-5D-3L index scores than their better educated counterparts. Higher levels of education were found to be associated with lower percentages of people reporting problems in all of the five health dimensions of EQ-5D-3L in the bivariate analyses and in mobility, pain/discomfort, and anxiety/depression in the logistic regression models. This is consistent with the results reported in Sri Lanka [39]. Poverty is another significant predictor of low HRQOL. Those living in poverty have an OR around 2 for reporting health problems. Indeed, poverty has been considered as an independent important factor affecting people’s HRQOL [24,63,64,65,66,67]. People with lower incomes have been reported with lower health status than those with higher incomes [31,37,39]. Consistent with findings in Singapore [37,38] and Sri Lanka [39], unemployed and retired people were found to be more likely to report health problems and have significantly lower ED-5Q-3L index scores than those who were employed. These results are also supported by a national study in China [24].

In our multivariate regression models, health insurance was statistically insignificant in predicting ED-5Q-3L index scores and reported health problems, and residency remained a significant predictor of reported health problems, but it became statistically insignificant in predicting ED-5Q-3L index scores. This does not necessarily indicate a lack of differences in health between urban and rural residents and between those with and without insurance coverage. As a matter of fact, urban residents and those covered by insurance are more likely to be wealthier and better educated. Those socioeconomic factors are often mingled together [68]. Such a phenomenon has significant policy implications. Social determinants of health have to be addressed in a systematic way. As is advocated by the World Health Organization, health has to be a focus in all policies [69,70]. Singling out one factor alone would not be able to offer an effective solution to population health improvement.

Our multivariate analyses found that people who engaged in regular physical activities had higher EQ-5D-3L scores. They were less likely to report problems in mobility, self-care, usual activities, and anxiety/depression. This has not been observed in previous studies [24,36,38,39,41]. It is not clear whether physical activities contribute to health or people with better health are more able to engage in physical activities. However, it is interesting to note that physical activities are not associated with reported pain/discomfort. Physical inactivity has been identified as the fourth leading risk factor for global mortality (6% of deaths globally) [71]. Regular moderate physical activities, such as walking and cycling, have significant benefits for health and can reduce the risks of coronary heart disease and stroke, diabetes, hypertension, colon cancer, breast cancer, and depression [71,72,73,74,75,76,77]. Unfortunately, participation in leisure time physical activities in China is low according to recommendations made by the World Health Organization [72].

This study has several limitations. Firstly, the survey involved a multi-stage sampling with both stratification and clustering, which makes it difficult to consider the clustering effects in data analyses. This might affect the precision of our estimates in the modeling. However, the representativeness of the sample has been widely accepted [42]. In addition, household members were interviewed separately, and we also excluded surrogate responses in our analyses. This can minimize the mutual influences among respondents within a household. Secondly, data used in this study did not have a perfect match with the assumptions of homoscedasticity and normality of the estimation errors, which is a common problem for the EQ-5D-3L. However, we still performed OLS regression analyses because the majority of EQ-5D-3L studies used OLS models [31,37,38,41] and a few studies comparing different modelling approaches recommend OLS [7,78,79]. The OLS model presented in this study can explain about 20% of the variances of the ED-5D index scores. Thirdly, it is important to note that participants of this study were restricted to those from one province; as such, caused must be exercised in generalizing the results. China is so large and diverse in culture and social development, and so regional population norms may be more appropriate than national norms. This study has, for the first time, provided EQ-5D-3L norms in China’s Heilongjiang province. The results revealed socioeconomic inequality in HRQOL. This has significant policy implications in relation to resource allocation. Further studies will be needed to explore regional differences in ED-5D-3L index scores.

## 5. Conclusions

This article provides important population norms for the EQ-5D-3L in Heilongjiang province of China for measuring health-related quality of life. These norms can be used to inform Chinese policymakers, health care professionals, and researchers in issues related to health care policy and planning. Health care needs are significant predictors of HRQOL. However, socioeconomic inequality in HRQOL is also evident. Priorities in health services should be given to those living with low socioeconomic status.

## Figures and Tables

**Table 1:** Characteristics of respondents and EuroQol five-dimension three-level (EQ-5D-3L) index and visual analogue scale (VAS) scores.

Characteristics of Respondents	EQ-5D-3L Index					EQ-5D VAS			
	N (%)	Mean (SD)	Ceiling Effect ^††^ (%)	*p (t/F)*	*p (M-W/K-W)*	Mean (SD)	Ceiling Effect ^††^ (%)	*p (t/F)*	*p (M-W/K-W)*
***Total***	11523 (100.0)	0.959 (0.124)	85.05			79.60 (15.70)	12.51		
***Age (year)***									
18–29	1742 (15.1)	0.993 (0.053)	97.47			88.42 (10.22)	23.25		
30–39	2467 (21.4)	0.982 (0.080)	91.93			84.43 (12.97)	17.67		
40–49	2862 (24.8)	0.970 (0.093)	86.37			80.43 (14.65)	12.33		
50–59	2494 (21.6)	0.952 (0.132)	81.68			75.74 (15.92)	7.42		
60–69	1222 (10.6)	0.912 (0.184)	71.69			70.99 (16.35)	4.09		
70+	736 (6.4)	0.865 (0.220)	61.01	0.000	0.000	66.73 (17.57)	1.77	0.000	0.000
***Sex***									
Male	5628 (48.8)	0.962 (0.122)	86.50			80.57 (15.41)	13.66		
Female	5895 (51.2)	0.957 (0.126)	83.66	0.033	0.000	78.68 (15.92)	11.42	0.000	0.000
***Ethnicity***									
Han	10973 (95.2)	0.960 (0.123)	85.17			79.58 (15.68)	12.30		
Others	550 (4.8)	0.948 (0.148)	82.55	0.053	0.063	80.13 (16.11)	16.73	0.424	0.239
***Residence***									
Urban	4631 (40.2)	0.955 (0.131)	83.01			78.49 (15.59)	8.83		
Rural	6892 (59.8)	0.962 (0.120)	86.42	0.001	0.000	80.35 (15.73)	14.99	0.000	0.000
***Education***									
Below primary school	931 (8.1)	0.896 (0.191)	65.95			68.47 (17.89)	4.08		
Primary school	3037 (26.4)	0.944 (0.145)	80.77			76.92 (16.38)	10.11		
Junior middle school	5339 (46.3)	0.971 (0.105)	88.35			81.39 (14.69)	14.67		
Senior middle school	1693 (14.7)	0.977 (0.089)	90.31			83.12 (13.67)	15.00		
College and above	523 (4.5)	0.988 (0.055)	93.12	0.000	0.000	85.39 (11.36)	11.47	0.000	0.000
***Housing***									
Flat/apartment	2196 (19.1)	0.962 (0.123)	85.56			80.08 (14.77)	8.24		
Brick bungalow	6697 (58.1)	0.962 (0.120)	86.02			80.13 (15.37)	14.02		
Mud-brick bungalow	2556 (22.2)	0.951 (0.135)	82.63			77.89 (17.16)	12.32		
Other ^†^	74 (0.6)	0.902 (0.171)	64.86	0.000	0.000	77.09 (15.31)	9.46	0.000	0.000
***Officially recorded poverty***									
Yes	1390 (12.1)	0.910 (0.175)	69.42			72.44 (18.36)	7.70		
No	10133 (87.9)	0.966 (0.114)	87.19	0.000	0.000	80.59 (15.04)	13.17	0.000	0.000
***Marital status***									
Never married	818 (7.1)	0.989 (0.072)	95.84			88.28 (11.45)	24.82		
Married	9905 (86.0)	0.961 (0.120)	85.38			79.57 (15.49)	12.08		
Divorced	182 (1.6)	0.962 (0.094)	81.87			79.16 (14.86)	9.34		
Widowed	618 (5.4)	0.888 (0.203)	66.34	0.000	0.000	68.77 (17.17)	4.05	0.000	0.000
***Employment***									
Employed	7287 (63.2)	0.975 (0.091)	89.65			82.13 (14.55)	15.74		
Retired	1266 (11.0)	0.930 (0.169)	76.15			74.15 (15.86)	4.66		
Unemployed	2970 (25.8)	0.932 (0.161)	77.54	0.000	0.000	75.73 (16.93)	7.95	0.000	0.000
***Health insurance***									
Yes	8548 (74.2)	0.962 (0.121)	85.86			80.03 (15.56)	13.27		
No	2975 (25.8)	0.952 (0.135)	82.72	0.000	0.000	78.38 (16.03)	10.35	0.000	0.000
***Chronic conditions (over the past six months)***									
Yes	2517 (21.8)	0.872 (0.208)	57.77			66.85 (17.22)	2.15		
No	9006 (78.2)	0.984 (0.071)	92.67	0.000	0.000	83.17 (13.21)	15.41	0.000	0.000
***Regular weekly physical activities***									
Yes	2160 (18.7)	0.965 (0.095)	83.43			79.60 (14.58)	9.86		
No	9363 (81.3)	0.958 (0.130)	85.42	0.058	0.084	79.61 (15.95)	13.13	0.990	0.216

^†^ having no house or no permanent accommodation. SD: standard deviation. *p (t/F): p* values of *t* tests or ANOVA tests; *p (M-W/K-W): p* values of Mann-Whitney U tests or Kruskal-Wallis tests. ^††^ Ceiling effect indicates the proportion of respondents with the best possible theoretical scores [48].

**Table 2:** Percentage of reported problems in five health dimensions of EQ-5D-3L (N = 11,523).

	Mobility			Self-Care			Usual Activities			Pain/Discomfort			Anxiety/Depression		
	No %/N	Some %/N	Extreme %/N	No %/N	Some %/N	Extreme %/N	No %/N	Some %/N	Extreme %/N	No %/N	Some %/N	Extreme %/N	No %/N	Some %/N	Extreme %/N
***Total***	94.6	4.9	0.5	96.3	3.1	0.5	94.7	4.5	0.9	87.9	11.4	0.7	92.8	6.6	0.6
***Sex***															
Male	94.2	5.3	0.5	96.2	3.2	0.6	94.6	4.5	1.0	***89.5***	***10.0***	***0.5***	***93.8***	***5.7***	***0.5***
Female	94.9	4.6	0.5	96.5	3.0	0.5	94.7	4.5	0.8	***86.4***	***12.7***	***0.9***	***91.8***	***7.5***	***0.7***
***Age (year)***															
18–29	***99.1***	***0.7***	***0.1***	***99.4***	***0.6***	***0.0***	***99.1***	***0.7***	***0.2***	***98.4***	***1.5***	***0.1***	***98.6***	***1.3***	***0.2***
30–39	***98.1***	***1.7***	***0.3***	***99.1***	***0.8***	***0.2***	***98.1***	***1.6***	***0.3***	***93.8***	***5.9***	***0.3***	***96.1***	***3.5***	***0.4***
40–49	***96.8***	***2.9***	***0.3***	***98.2***	***1.6***	***0.2***	***97.2***	***2.4***	***0.4***	***89.8***	***10.0***	***0.2***	***93.3***	***6.4***	***0.3***
50–59	***93.6***	***5.9***	***0.5***	***95.9***	***3.5***	***0.6***	***94.1***	***4.9***	***1.0***	***84.7***	***14.4***	***0.9***	***91.9***	***7.5***	***0.7***
60–69	***88.1***	***10.7***	***1.2***	***92.0***	***6.3***	***1.7***	***87.3***	***10.2***	***2.5***	***75.2***	***22.8***	***2.0***	***85.6***	***12.8***	***1.6***
70+	***77.3***	***21.1***	***1.6***	***81.7***	***15.9***	***2.4***	***76.8***	***19.8***	***3.4***	***68.1***	***29.2***	***2.7***	***81.4***	***17.0***	***1.6***
***Ethnicity***															
Han	***94.7***	***4.8***	***0.5***	***96.4***	***3.0***	***0.5***	***94.8***	***4.4***	***0.8***	***88.0***	***11.3***	***0.7***	92.8	6.6	0.6
Others	***91.3***	***7.6***	***1.1***	***94.7***	***4.4***	***0.9***	***92.5***	***5.8***	***1.6***	***86.2***	***13.3***	***0.5***	93.1	5.6	1.3
***Residence***															
Urban	94.1	5.4	0.5	96.0	3.2	0.8	94.6	4.4	1.0	***87.1***	***12.0***	***0.9***	***91.2***	***8.0***	***0.8***
Rural	94.9	4.6	0.5	96.6	3.0	0.4	94.7	4.5	0.8	***88.5***	***10.9***	***0.6***	***93.9***	***5.6***	***0.5***
***Education***															
Below primary school	***84.3***	***14.6***	***1.1***	***88.5***	***10.1***	***1.4***	***84.6***	***13.2***	***2.1***	***71.3***	***26.2***	***2.5***	***83.7***	***14.8***	***1.5***
Primary school	***92.4***	***6.9***	***0.7***	***94.7***	***4.5***	***0.8***	***91.7***	***6.9***	***1.4***	***83.7***	***15.3***	***0.9***	***90.6***	***8.6***	***0.8***
Junior middle school	***96.3***	***3.3***	***0.4***	***97.7***	***1.9***	***0.4***	***96.7***	***2.7***	***0.6***	***90.9***	***8.6***	***0.5***	***94.4***	***5.1***	***0.5***
Senior middle school	***97.3***	***2.5***	***0.2***	***98.2***	***1.5***	***0.2***	***97.5***	***2.2***	***0.3***	***92.9***	***6.8***	***0.4***	***95.4***	***4.3***	***0.4***
**College and above**	***98.7***	***1.3***	***0.0***	***99.6***	***0.2***	***0.2***	***99.2***	***0.4***	***0.4***	***95.8***	***4.2***	***0.0***	***97.1***	***2.9***	***0.0***
***Housing***															
Flat/apartment	***95.1***	***4.3***	***0.5***	***97.0***	***2.2***	***0.8***	***96.1***	***3.1***	***0.9***	***89.1***	***10.0***	***0.9***	***92.9***	***6.5***	***0.6***
Brick bungalow	***94.8***	***4.7***	***0.5***	***96.6***	***2.9***	***0.5***	***94.9***	***4.2***	***0.8***	***88.9***	***10.5***	***0.6***	***93.3***	***6.2***	***0.6***
Mud-brick bungalow	***93.6***	***5.9***	***0.5***	***95.2***	***4.3***	***0.5***	***92.9***	***6.2***	***0.9***	***84.7***	***14.4***	***0.9***	***91.8***	***7.6***	***0.6***
Other ^†^	***90.5***	***9.5***	***0.0***	***94.6***	***5.4***	***0.0***	***87.8***	***9.5***	***2.7***	***73.0***	***23.0***	***4.1***	***81.1***	***12.2***	***6.8***
***Officially recorded poverty***															
Yes	***87.0***	***12.3***	***0.7***	***90.9***	***7.8***	***1.3***	***87.1***	***11.2***	***1.8***	***76.8***	***21.9***	***1.4***	***82.2***	***16.0***	***1.9***
No	***95.6***	***3.9***	***0.5***	***97.1***	***2.5***	***0.4***	***95.7***	***3.6***	***0.7***	***89.5***	***9.9***	***0.6***	***94.3***	***5.3***	***0.5***
***Marital status***															
Never married	***98.5***	***1.2***	***0.2***	***99.0***	***0.7***	***0.2***	***98.7***	***0.9***	***0.5***	***97.3***	***2.6***	***0.1***	***98.2***	***1.5***	***0.4***
Married	***94.9***	***4.6***	***0.5***	***96.7***	***2.8***	***0.5***	***95.1***	***4.2***	***0.8***	***88.2***	***11.1***	***0.6***	***93.0***	***6.4***	***0.6***
Divorced	***96.7***	***3.3***	***0.0***	***96.7***	***3.3***	***0.0***	***96.2***	***3.8***	***0.0***	***85.7***	***14.3***	***0.0***	***90.1***	***9.9***	***0.0***
Widowed	***82.8***	***16.5***	***0.6***	***87.4***	***10.4***	***2.3***	***82.4***	***14.6***	***3.1***	***71.2***	***25.4***	***3.4***	***83.5***	***14.7***	***1.8***
***Employment***															
Employed	***97.1***	***2.6***	***0.3***	***98.2***	***1.7***	***0.2***	***97.0***	***2.6***	***0.4***	***91.4***	***8.3***	***0.3***	***95.6***	***4.1***	***0.3***
Retired	***89.5***	***9.6***	***0.9***	***93.1***	***5.4***	***1.5***	***90.4***	***7.6***	***2.1***	***81.0***	***17.5***	***1.6***	***89.7***	***9.1***	***1.2***
Unemployed	***90.5***	***8.8***	***0.8***	***93.2***	***5.7***	***1.1***	***90.8***	***7.6***	***1.6***	***82.4***	***16.3***	***1.3***	***87.1***	***11.6***	***1.2***
***Medical insurance***															
Yes	***94.8***	***4.7***	***0.5***	***96.6***	***3.0***	***0.5***	***94.9***	***4.3***	***0.8***	***88.4***	***11.0***	***0.6***	***93.6***	***5.8***	***0.6***
No	***93.8***	***5.6***	***0.5***	***95.7***	***3.5***	***0.8***	***93.9***	***5.1***	***0.9***	***86.6***	***12.4***	***0.9***	***90.5***	***8.7***	***0.8***
***Chronic conditions (over the past six months)***															
Yes	***82.8***	***15.5***	***1.7***	***87.6***	***10.4***	***2.0***	***82.0***	***14.9***	***3.0***	***63.2***	***33.9***	***2.9***	***79.0***	***18.7***	***2.3***
No	***97.8***	***2.0***	***0.1***	***98.8***	***1.1***	***0.1***	***98.2***	***1.6***	***0.3***	***94.9***	***5.0***	***0.1***	***96.7***	***3.2***	***0.2***
***Regular weekly physical activities***															
Yes	***96.0***	***3.9***	***0.1***	***98.0***	***1.8***	***0.2***	***96.0***	***3.7***	***0.3***	87.1	12.5	0.3	93.0	6.8	0.3
No	***94.2***	***5.2***	***0.6***	***96.0***	***3.4***	***0.6***	***94.3***	***4.7***	***1.0***	88.1	11.1	0.8	92.8	6.5	0.7

Note: Numbers with bold italic font indicate *p <* 0.05; Numbers with underlined bold italic font indicate *p <* 0.01. **^†^** having no house or permanent accommodation.

**Table 3:** Associations between characteristics and EQ-5D-3L index and its health problems reported in five dimensions.

	Mobility OR (95% CI)	Self-Care OR (95% CI)	Usual Activities OR (95% CI)	Pain/Discomfort OR (95% CI)	Anxiety/Depression OR (95% CI)	EQ-5D-3L Index Beta (95% CI)
***Sex***						
Male	Reference	Reference	Reference	Reference	Reference	Reference
Female	0.6 (0.5, 0.7) **	0.7 (0.5, 0.9) **	0.7 (0.6, 0.9) **	1.1 (1.0, 1.3)	1.1 (0.9, 1.3)	0.00 (0.00, 0.01)
***Age (year)***						
18–29	Reference	Reference	Reference	Reference	Reference	Reference
30–39	1.7 (0.9, 3.2)	1.0 (0.5, 2.2)	1.4 (0.8, 2.6)	2.8 (1.8, 4.4) **	1.8 (1.1, 2.9) *	0.00 (−0.01, 0.01)
40–49	2.0 (1.1, 3.7) *	1.4 (0.7, 2.8)	1.5 (0.8, 2.7)	3.7 (2.4, 5.6) **	2.4 (1.5, 3.8) **	0.00 (−0.01, 0.01)
50–59	3.1 (1.7, 5.5) **	2.5 (1.3, 5.0) **	2.3 (1.3, 4.1) **	4.3 (2.8, 6.5) **	2.3 (1.4, 3.6) **	−0.01 (−0.02, 0.00) *
60–69	3.4 (1.8, 6.4) **	3.0 (1.5, 6.2) **	3.2 (1.7, 5.8) **	4.8 (3.0, 7.5) **	2.6 (1.6, 4.3) **	−0.03 (−0.04, −0.02) **
70+	5.9 (3.1, 11.1) **	6.7 (3.2, 14.2)	5.7 (3.0, 10.7) **	5.7 (3.5, 9.3) **	2.9 (1.7, 4.9) **	−0.06 (−0.07, −0.05) **
***Ethnicity***						
Han	Reference	Reference	Reference	Reference	Reference	Reference
Others	1.6 (1.1, 2.3) **	0.7 (0.5, 1.1)	1.3 (0.9, 1.9)	1.1 (0.9, 1.5)	0.9 (0.6, 1.3)	−0.01 (−0.02, −0.00)
***Residence***						
Urban	Reference	Reference	Reference	Reference	Reference	Reference
Rural	1.6 (1.2, 2.3) **	1.2 (0.8, 1.8)	1.8 (1.3, 2.5) **	1.2 (0.9, 1.5)	1.0 (0.7, 1.3)	0.01 (0.00, 0.01)
***Education***						
Below primary school	Reference	Reference	Reference	Reference	Reference	Reference
Primary school	1.0 (0.8, 1.3)	1.1 (0.8, 1.5)	1.3 (1.0, 1.7)	0.9 (0.8, 1.2)	1.0 (0.8, 1.3)	0.00 (0.00, 0.01)
Junior middle school	0.7 (0.5, 0.9) *	0.8 (0.6, 1.2)	0.8 (0.6, 1.1)	0.7 (0.6, 0.9) **	0.8 (0.6, 1.0)	0.01 (0.00, 0.02) **
Senior middle school	0.6 (0.4, 0.9) *	0.8 (0.5, 1.2)	0.8 (0.5, 1.2)	0.6 (0.5, 0.8) **	0.7 (0.5, 0.9) *	0.01 (0.00, 0.02) *
College and above	0.4 (0.2, 1.0)	0.3 (0.1, 1.1)	0.4 (0.1, 1.1)	0.5 (0.3, 0.8) **	0.6 (0.3, 1.1)	0.01 (0.00, 0.03)
***Housing***						
Flat/apartment	Reference	Reference	Reference	Reference	Reference	Reference
Brick bungalow	0.9 (0.7, 1.2)	1.0 (0.7, 1.4)	1.2 (0.9, 1.6)	1.1 (0.9, 1.4)	1.0 (0.7, 1.2)	0.00 (0.00, 0.01)
Mud-brick bungalow	0.9 (0.6, 1.3)	1.2 (0.8, 1.9)	1.3 (0.9, 2.0)	1.4 (1.1, 1.8) *	1.1 (0.8, 1.5)	0.00 (−0.01, 0.01)
Other ^†^	1.3 (0.5, 3.2)	1.2 (0.4, 3.7)	2.4 (1.0, 5.7) *	3.2 (1.7, 6.0) **	2.8 (1.4, 5.6) **	−0.04 (−0.07, −0.02) **
***Officially recorded poverty***						
No	Reference	Reference	Reference	Reference	Reference	Reference
Yes	2.4 (1.9, 3.0) **	2.2 (1.7, 2.8) **	2.5 (2.0, 3.1) **	2.0 (1.7, 2.4) **	2.6 (2.1, 3.1) **	−0.04 (−0.04, −0.03) **
***Marital status***						
Never married	Reference	Reference	Reference	Reference	Reference	Reference
Married	1.2 (0.6, 2.3)	1.1 (0.5, 2.5)	1.3 (0.7, 2.6)	1.4 (0.8, 2.2)	1.5 (0.8, 2.6)	0.00 (−0.01, 0.01)
Divorced	0.7 (0.2, 2.1)	1.3 (0.4, 4.2)	1.1 (0.4, 3.0)	1.8 (0.9, 3.5)	1.7 (0.8, 3.7)	0.00 (−0.02, 0.02)
Widowed	1.4 (0.7, 2.8)	1.2 (0.5, 2.9)	1.5 (0.7, 3.2)	1.4 (0.8, 2.4)	1.4 (0.7, 2.6)	−0.01 (−0.02, 0.00)
***Employment***						
Employed	Reference	Reference	Reference	Reference	Reference	Reference
Retired	3.3 (2.3, 4.8) **	2.7 (1.7, 4.1) **	3.0 (2.0, 44) **	1.6 (1.2, 2.1) **	1.6 (1.2, 2.2) **	−0.02 (−0.02, −0.01) **
Unemployed	2.7 (2.1, 3.5) **	2.4 (1.8, 3.3) **	2.4 (1.8, 3.1) **	1.6 (1.4, 2.0) **	2.2 (1.7, 2.7) **	−0.03 (−0.03, 0.02) **
***Health insurance***						
No	Reference	Reference	Reference	Reference	Reference	Reference
Yes	1.0 (0.7, 1.2)	1.0 (0.7, 1.4)	0.9 (0.7, 1.1)	1.1 (0.9, 1.3)	1.0 (0.8, 1.3)	0.00 (0.00, 0.01)
***Chronic conditions***						
No	Reference	Reference	Reference	Reference	Reference	Reference
Yes	6.0 (5.0, 7.3) **	6.9 (5.4, 8.8) **	7.6 (6.2, 9.2) **	7.4 (6.5, 8.5) **	5.6 (4.7, 6.6) **	−0.09 (−0.10, −0.09) **
***Regular weekly physical activities***						
No	Reference	Reference	Reference	Reference	Reference	Reference
Yes	0.4 (0.3, 0.6) **	0.3 (0.2, 0.4) **	0.5 (0.4, 0.6) **	1.0 (0.8, 1.2)	0.8 (0.6, 1.0) *	0.02 (0.01, 0.02) **
**Cox and Snell R^2^**	0.096	0.079	0.104	0.155	0.090	
Nagelkerke R^2^	0.278	0.293	0.306	0.297	0.222	
R^2^						0.186
Adjusted R^2^						0.184

* *p* < 0.05; ** *p* < 0.01. ^†^ having no house or permanent accommodation. OR: odds ratio. CI: confidence interval.

## Supplementary Materials

The following are available online at www.mdpi.com/1660-4601/14/3/224/s1, Table S1: Factors associated with EQ-5D index scores—results of Tobit regression analyses.
