# Supplementary material for: Assessing Health-Related Quality of Life of Chinese Adults in Heilongjiang Using EQ-5D-3L
Source: Int J Environ Res Public Health. 2017 Feb 23;14(3):224. doi: 10.3390/ijerph14030224 (PMC5369060; doi:10.3390/ijerph14030224)
Supplement: Supplementary file 1 [file ijerph-14-00224-s001.doc]

Table S1. Factors associated with EQ-5D index scores—results of Tobit regression analyses

| **EQ-5D-3L Index** | **Coef.** | **Std. Err.** | **t** | **P > t** | **[95% Conf. Interval]** | |
| --- | --- | --- | --- | --- | --- | --- |
| **Sex (male)** |  |  |  |  |  |  |
| **Female** | 0.007 | 0.013 | 0.52 | 0.606 | −0.018 | 0.031 |
| **Age (18–29)** |  |  |  |  |  |  |
| **30–39** | −0.152 | 0.032 | −4.72 | *0.000* | −0.216 | −0.089 |
| **40–49** | −0.205 | 0.032 | −6.46 | *0.000* | −0.267 | −0.143 |
| **50–59** | −0.232 | 0.032 | −7.17 | *0.000* | −0.296 | −0.169 |
| **60–69** | −0.261 | 0.036 | −7.32 | *0.000* | −0.331 | −0.191 |
| **70+** | −0.331 | 0.040 | −8.38 | *0.000* | −0.409 | −0.254 |
| **Ethnicity (Han)** |  |  |  |  |  |  |
| **Others** | −0.044 | 0.027 | −1.6 | 0.109 | −0.098 | 0.010 |
| **Residence (Urban)** |  |  |  |  |  |  |
| **Rural** | 0.018 | 0.022 | 0.81 | 0.417 | −0.025 | 0.061 |
| **Education** |  |  |  |  |  |  |
| **Primary school** | 0.014 | 0.021 | 0.67 | 0.503 | −0.027 | 0.056 |
| **Junior middle school** | 0.075 | 0.023 | 3.28 | *0.001* | 0.030 | 0.120 |
| **Senior middle school** | 0.111 | 0.029 | 3.84 | *0.000* | 0.054 | 0.167 |
| **College and above** | 0.110 | 0.045 | 2.46 | *0.014* | 0.022 | 0.197 |
| **Housing (Flat/apartment)** |  |  |  |  |  |  |
| **Brick bungalow** | −0.002 | 0.020 | −0.08 | 0.935 | −0.041 | 0.038 |
| **Mud-brick bungalow** | −0.029 | 0.025 | −1.16 | 0.247 | −0.079 | 0.020 |
| **Other†** | −0.227 | 0.062 | −3.64 | *0.000* | −0.349 | −0.105 |
| **Officially recorded poverty (No)** |  |  |  |  |  |  |
| **Yes** | −0.185 | 0.016 | −11.23 | *0.000* | −0.217 | −0.153 |
| **Marital status (Never married)** |  |  |  |  |  |  |
| **Married** | −0.032 | 0.039 | −0.82 | 0.413 | −0.107 | 0.044 |
| **Divorced** | −0.041 | 0.060 | −0.67 | 0.500 | −0.158 | 0.077 |
| **Widowed** | −0.044 | 0.045 | −0.98 | 0.327 | −0.132 | 0.044 |
| **Employment (Employed)** |  |  |  |  |  |  |
| **Retired** | −0.120 | 0.026 | −4.66 | *0.000* | −0.170 | −0.069 |
| **Unemployed** | −0.135 | 0.018 | −7.39 | *0.000* | −0.170 | −0.099 |
| **Health insurance (No)** |  |  |  |  |  |  |
| **Yes** | 0.009 | 0.017 | 0.52 | 0.601 | −0.024 | 0.042 |
| **Chronic conditions (No)** |  |  |  |  |  |  |
| **Yes** | −0.413 | 0.014 | −28.76 | *0.000* | −0.441 | −0.385 |
| **Regular weekly physical activities (No)** | |  |  |  |  |  |
| **Yes** | 0.042 | 0.018 | 2.34 | *0.020* | 0.007 | 0.077 |
| **LR** | 2280 |  |  |  |  |  |

^†^having no house or permanent accommodation.
